# When AI simulates teaching personas: Linguistic dimensions of LLM-generated classroom discourse

**DOI:** 10.1371/journal.pone.0360650

**Published:** 2026-11-06

**Authors:** Tony Berber Sardinha, Marilisa Shimazumi

**Affiliations:** 1 Graduate Program in Applied Linguistics and Language Studies (LAEL) & Graduate Program in Social Psychology (PSO)/Pontifical Catholic University of Sao Paulo, Brazil; 2 Sao Paulo State University at S. José do Rio Preto (UNESP-SJRP), Brazil; Shibaura Institute of Technology, JAPAN

## Abstract

This study investigates how GPT-4o constructs the social role of teacher through discourse and how this discourse compares with naturally occurring classroom teaching. We generated 19,950 AI classroom teaching texts from a corpus of online college teaching using 56 teacher personas plus a baseline condition. The AI-generated texts contained the original student turns alongside the AI-teacher turns. We analyzed the resulting corpus using Lexical Multi-Dimensional Analysis (LMDA), a bottom-up corpus-linguistic method that identifies dimensions of discourse variation from recurrent patterns of lexical covariation. Five dimensions emerged: Macro-Structuring Discourse vs. Performative Stagecraft, L1-scaffolded Guidance vs. Provocative Challenge, Procedural Control vs. Rapport-driven Engagement, Evaluative Control vs. Reflective Aesthetic Engagement, and Informal Facilitation vs. Formal Deductive Exposition. Comparison with the corresponding human classroom discourse showed that GPT-4o frequently magnified teaching discourses already present in human interaction, particularly organizational, procedural, and expository discourse. It also introduced more artificial forms of teaching discourse characterized by theatrical performance, confrontation, and evaluative pressure. Mixed-effects analyses showed significant differences between human and AI discourse on all five dimensions. Persona prompting also affected dimension scores, but differentiation among the 57 conditions was uneven: many personas occupied overlapping positions, and differences occurred on only some dimensions. The findings show that GPT-4o can produce distinct forms of teaching discourse, yet many different persona prompts repeat similar discourses, suggesting a saturation point beyond which the LLM cannot yield new teaching discourses. More generally, the study demonstrates how corpus-based discourse analysis can characterize the forms of teaching that emerge when an LLM is instructed to perform a professional role.

Teacher: And what did you do at the weekend, Diego? Did you go to the park? Did you go to the shops? Did you dress up as an astronaut and go to the Moon?

Student: No.

Teacher: No what?

Student: I did not go to the Moon.

Teacher: That was me being sarcastic.

(The Penguin Lessons [movie], 2024)

## Introduction

The present study investigates the discourse through which GPT-4o constructs the social role of teacher. Rather than asking whether the model teaches effectively or accurately reproduces classroom practice, we examine the discourse repertoire that becomes available when GPT-4o is instructed to act as a teacher. Specifically, we ask what discourse dimensions characterize this repertoire, how these dimensions relate to naturally occurring teacher discourse, and how they are affected by extensive persona variation.

One way of sampling this repertoire is through persona prompting. In LLM research, personas specify characteristics that the model is instructed to adopt during generation, including profession, personality, expertise, ideology, demographic background, or interpersonal style. Rather than viewing personas as fixed identities that a model either reproduces successfully or fails to reproduce, we treat them as methodological probes that expose different regions of the discourse repertoire associated with a single social role.

Early work introduced personas as a means of personalizing dialog systems [1]. More recent studies have examined persona prompting for applications including conversation generation, social simulation, fairness, evaluation, and domain-specific performance [2–5]. Most of this work evaluates whether personas alter model behavior or improve performance. Much less attention has been given to the linguistic organization of the discourse generated under persona variation.

This gap is particularly evident for the teacher role. Although teachers have occasionally appeared among broader collections of occupational personas [5,6], previous studies have not investigated the discourse system through which LLMs construct teaching. Consequently, little is known about the range of teaching discourses encoded in these models or about the extent to which persona variation expands or reorganizes this repertoire.

To investigate this problem, we generated classroom discourse using GPT-4o under 57 teacher persona conditions varying personality traits, pedagogical orientations, and social characteristics. The objective was to sample as broadly as possible an LLM’s discourse repertoire associated with the teacher role.

The resulting corpus was analyzed using Lexical Multi-Dimensional Analysis (LMDA) [7], a bottom-up corpus linguistic method that identifies discourse dimensions from patterns of lexical co-occurrence. Unlike approaches that classify outputs according to predefined personality, occupational, or pedagogical categories, LMDA derives the principal dimensions of discourse directly from the linguistic evidence.

Human classroom discourse served as a reference for interpreting the dimensions. The comparison is not intended to assess whether GPT-4o reproduces authentic teaching or to evaluate its effectiveness as an instructional system. Rather, it provides an empirical basis for situating AI-generated teaching discourse within the broader space of naturally occurring classroom discourse. Accordingly, the purpose of this study is to characterize the discourse repertoire through which GPT-4o constructs the teacher role under extensive persona variation. Specifically, we investigate whether diverse persona prompts produce an expanding range of teaching discourses or whether they converge on a smaller set of recurrent discourse dimensions.

The following research questions guide the analysis:

RQ1 What are the major discourse dimensions through which GPT-4o constructs the teacher role?
RQ2 How are these AI-generated teaching discourses positioned relative to human classroom discourse?
RQ3 To what extent do teacher personas differ in their positions on the discourse dimensions identified by LMDA?

The remainder of the paper reviews previous research on persona prompting and LLM role simulation before presenting the corpus, the multidimensional analysis, and the discourse dimensions that characterize the realization of the teacher role by GPT-4o.

## Representations of the teacher role

To sample the LLM discourse repertoire, the persona prompts must vary the way the teacher role is represented. Educational research provides a rich source of such representations, having described teaching through multiple theoretical constructs including styles, perspectives, roles, and personality traits. We use these classifications not as taxonomies of real teachers that GPT-4o is expected to reproduce, but as sources of variation for probing the discourse repertoire of the model.

One influential tradition characterizes teaching in terms of instructional styles. For example, Grasha (1996) distinguishes five overlapping styles representing different emphases on disciplinary expertise, classroom authority, modeling, guidance, and learner autonomy – these are Expert, Formal Authority, Personal Model, Facilitator, and Delegator [8]. Other frameworks describe teaching through dimensions such as direct versus indirect instruction [9,10], classroom authority and responsiveness [11,12], or interpersonal relations [13]. Although these classifications differ theoretically, they converge in portraying the teacher role through recurring concerns such as expertise, control, interaction, and learner independence.

A second tradition focuses on broader teaching perspectives. Pratt et al. (1998) identifies five perspectives: Transmission, Apprenticeship, Developmental, Nurturing, and Social Reform [14], which differ in how they conceptualize the purposes of teaching, the nature of knowledge, and relationships between teachers and learners. Rather than describing classroom techniques, these perspectives represent alternative conceptions of what teaching is intended to accomplish.

Research has also characterized teachers through personality and interpersonal traits. Studies describe teachers in terms of dimensions such as warmth, organization, fairness, patience, enthusiasm, authority, and openness [15–18]. Other work emphasizes students’ perceptions of desirable teacher characteristics, including respectfulness, emotional stability, conscientiousness, and supportiveness [19–21]. Together, these studies show that the teacher role has been represented through cognitive, interpersonal, organizational, emotional, and social attributes.

Importantly, these classifications were developed for educational research rather than for describing discourse. They therefore provide independent ways of varying representations of the teacher role without imposing linguistic categories on the analysis itself. In the present study, these representations are used exclusively to construct persona prompts. The discourse dimensions are subsequently identified inductively through Lexical Multi-Dimensional Analysis, allowing the linguistic organization of the teaching discourse by GPT 4o to emerge bottom-up from the corpus rather than top-down from predefined educational labels.

## Corpus linguistics and lexical multi-dimensional analysis

Corpus Linguistics studies language through large collections of naturally occurring texts, or corpora, allowing linguistic regularities to be identified empirically rather than inferred from intuition or isolated examples [22–25]. By examining thousands or millions of words simultaneously, corpus analysis reveals recurring lexical and grammatical patterns that characterize how language is actually used across texts.

In this tradition, corpora have become an important resource for discourse analysis. Corpus-assisted discourse analysis (CADS) identifies linguistic patterns that recur throughout an entire corpus, reducing the possibility of selective interpretation [26,27]. Discourse is understood here as socially shared, socially situated representations of real-world phenomena conveyed implicitly through language use [7]. Under this view, discourses emerge from recurring configurations of shared linguistic features distributed across texts.

Traditional CADS studies typically investigate these configurations using methods such as frequency analysis, concordances, collocations, and keyword analysis. These techniques have proved effective for identifying salient linguistic patterns, but these patterns reflect frequency differences rather than covariation among linguistic features. The present study instead adopts Lexical Multi-Dimensional Analysis, which identifies underlying discourse structures statistically through patterns of covariation. Covariation helps identify sets of linguistic features that are meaningful because they tend to occur together or because they tend to occur apart. Thus, discourse dimensions reflect patterns of both co-occurrence and mutual exclusion.

Lexical Multi-Dimensional Analysis (LMDA) [7] extends the principles of Multi-Dimensional Analysis [28] to the lexical level. Like its predecessor, LMDA treats the text, rather than the individual word, as the unit of analysis. Each text is represented by its lexical profile, and factor analysis is used to identify latent dimensions based on patterns of lexical covariance across the corpus. The dimensions represent recurring shared lexical sets that characterize how texts are constructed. In turn, these sets offer entry points into the discourse level, by enabling researchers to dig into the underlying layers of the text and see which belief systems, imaginaries, representations, worldviews, and other forms of collective thinking and social action are encoded in them.

LMDA differs from conventional corpus methods in two important respects. First, it models relationships among complete texts rather than aggregating word frequencies across an entire corpus, thereby preserving discourse variation at the text level. Second, the discourse dimensions are derived inductively from the corpus itself rather than imposed through predefined categories. Interpretation therefore follows, rather than precedes, the statistical analysis: lexical features with the highest factor loadings are examined together with high-scoring texts to determine the discourse represented by each dimension.

The approach has been applied to a wide range of discourse domains, including language policy, migration, political communication, sustainability, religion, popular music, and academic writing [29–37]. In these studies, the general goal is to discover the principal dimensions organizing the discourse of the corpus under investigation.

LMDA is particularly appropriate for the present study because the teacher personas are not themselves the object of analysis. Rather, they are used to generate systematic variation in the corpus, allowing the underlying discourse repertoire associated with the teacher role to be sampled. The discourse dimensions are then identified directly from the linguistic evidence, enabling us to characterize how GPT-4o constructs the teacher role and to compare this repertoire with naturally occurring classroom discourse.

## Materials and methods

### Corpus design and generation

To sample the discourse repertoire associated with the teacher role, we developed 56 teacher personas based on the educational literature reviewed above together with our own teaching experience (Table 1). The personas varied representations of the teacher role along dimensions including instructional orientation, personality, interpersonal style, demographic characteristics, and identity. An additional baseline condition contained no teacher description, allowing GPT-4o to generate teacher discourse without explicit persona specification.

**Table 1:** Teacher personas.

Category	Personas
Affective and social behavior	anxious, assertive, calm, empathetic, enthusiastic, patient, sarcastic
Age	young, middle-aged, senior
Career stage	early-career, experienced
Ethnicity	Asian, Black, Indigenous, Latin American, White
Gender and sexual identity	female, LGBTQ, male, non-binary
Interactional style	teacher-centered, student-centered, interactive
L1 background	native English speaker, non-native English speaker
Pedagogical orientation	authoritative, behaviorist, constructivist, delegator, expert, facilitator, formal authority, humanistic, personal model, pragmatic, tech-enthusiast, traditionalist
Style	adaptive, awkward, cold, critical, encouraging, gentle, harsh, humble, methodical, passive, reflective, relaxed, Socratic, strict, supportive, warm
Verbal engagement	reserved, talkative

Teacher personas used in the study, grouped by pedagogical, demographic, social, and personal traits.

The human reference corpus consisted of transcriptions of authentic online classes taught at a private college (This study was approved by the corresponding institution's Ethics Committee in July 2025. The human classroom data were previously collected, fully anonymized, and made available for academic research. No identifiable information was available to the authors). Seven courses were included: two English language courses (B1–B2 and C1) and five graduate courses (Introduction to Bilingualism, Children’s Literature, Language Awareness, Literature and Its Media, and Language Policy).

After anonymization, transcripts were segmented into units of 20 consecutive turns. Segments containing between 8 and 18 teacher turns were retained, and 50 segments were randomly selected from each course, yielding a human corpus of 350 classroom segments.

Each human segment served as the basis for generating an AI counterpart. The complete prompt consisted of a system prompt defining the target teacher persona and reconstruction instructions, followed by two user messages. The first user message specified the instructional goal of the class, whereas the second contained the original classroom transcript. Within this transcript, the original student turns were preserved verbatim, while GPT-4o was instructed to replace only the teacher turns in a manner consistent with both the assigned persona and the students’ contributions. Thus, each prompt contained (i) the instructional goal, (ii) the original classroom transcript, (iii) the assigned teacher persona, and (iv) the reconstruction instructions. The complete prompt was archived for every generated text to ensure reproducibility. Responses were generated through the OpenAI Chat Completions API using GPT-4o. No explicit sampling parameters (e.g., temperature) were specified; consequently, the API default settings were used throughout. Generated outputs were automatically checked for completeness, and incomplete generations were discarded and regenerated.

The corpus was generated in March 2025 using GPT-4o because, at that time, it represented one of the most mature and extensively evaluated frontier language models available. Having undergone nearly ten months of iterative refinement since its release, GPT-4o offered a stable platform for large-scale text generation, with well-documented performance and predictable behavior. It also provided an advantageous balance between generation quality, computational efficiency, and operational cost, making it particularly suitable for producing the large corpus required in this study. Consequently, GPT-4o was selected not because it was assumed to be representative of all large language models, but because it constituted a reliable and practical state-of-the-art model at the time of data collection.

Table 2 illustrates this procedure by showing an original classroom segment, the interpretive cues supplied to GPT-4o, and the resulting AI-generated teacher discourse.

**Table 2:** Excerpt of a human teacher-led class segment, its corresponding AI generation cues, and the resulting AI-generated class segment (for the adaptive teacher profile).

Speaker	Human-Led Class Segment	AI Cues	AI-Generated Class Segment
[Speaker 1]	Yes. What else, guys, would you have to say about discourse? Because there is not only one definition to discourse.	The teacher opens the discussion, prompting the students to provide their own ideas of what the term ‘discourse’ means, as there’s no single definition.	You’ve made an excellent observation about how discourse shapes our understanding. Let’s consider other viewpoints—what else comes to mind when we define ‘discourse’?
[Speaker 1]	And <name>, yes, <name>, please go ahead.	The teacher invites a specific student (<name>) to contribute to the discussion, encouraging participation.	Remember, different scholars define discourse in distinct ways. <name>, what are your thoughts on this?
[Speaker 8]	Ok, so I think it’s a way of communication and manipulation too. You can manipulate someone by discourse.	Ok, so I think it’s a way of communication and manipulation too. You can manipulate someone by discourse.	Ok, so I think it’s a way of communication and manipulation too. You can manipulate someone by discourse.
[Speaker 1]	Ok.	The teacher acknowledges that Speaker 8’s definition of discourse involves it being both a means of communication and potential tool for manipulation.	I hear you – please say more about that.
[Speaker 8]	So it’s a way of communication.	So it’s a way of communication.	So it’s a way of communication.
[Speaker 1]	Ok. So for you, discourse has to do with the ways we communicate. And also through discourse, as you said, we can also manipulate people somehow.	The teacher reiterates Speaker 8’s idea, highlighting that discourse is about communication and, according to Speaker 8’s perspective, can also be used to manipulate.	Just to make sure I understand: you’re suggesting that discourse functions as communication but also has the power to manipulate audiences, right?

This excerpt illustrates a real human-led class segment, the corresponding prompt cues designed for the AI to simulate a particular teacher profile, and the resulting AI-generated segment.

The final corpus is summarized in Table 3.

**Table 3:** Corpus size.

Strata	Subcorpus	Text Count	Word Count
Source	AI	19,950	28,054,701
	Human	350	533,703
Course	Use of English Levels B1 & B2 (b1-b2)	2,900	3,194,331
	Use of English Level C1 (c1)	2,900	3,602,466
	Bilingualism (bil)	2,900	5,329,680
	Children’s Literature (chi)	2,900	3,919,932
	Language Awareness (law)	2,900	4,463,202
	Literature and Its Media (lit)	2,900	4,072,785
	Language Policy (lpo)	2,900	4,006,008
Course type	English Instruction	5,800	6,796,797
	Graduate Courses	14,500	21,791,607
Overall corpus		20,300	28,588,404

Corpus design showing the distribution of texts and word counts for the human-taught and AI-simulated texts.

### Lexical multi-dimensional analysis

The analysis was conducted exclusively on teacher turns extracted from both the human and AI corpora. Student turns were excluded from the statistical analysis but were retained during the interpretation of the resulting discourse dimensions.

Teacher turns were part-of-speech tagged and lemmatized using the TreeTagger [38]. Common nouns, lexical verbs, adjectives, adverbs, interjections, and foreign words were retained, and a binary lemma-by-text matrix was constructed.

Tetrachoric correlations among lemmas were computed in SAS and submitted to factor analysis. Inspection of the scree plot indicated a five-factor solution (Fig 1), which was subsequently rotated using Promax rotation. Only loadings with an absolute value of at least .30 were retained for interpretation.

**Fig 1:**
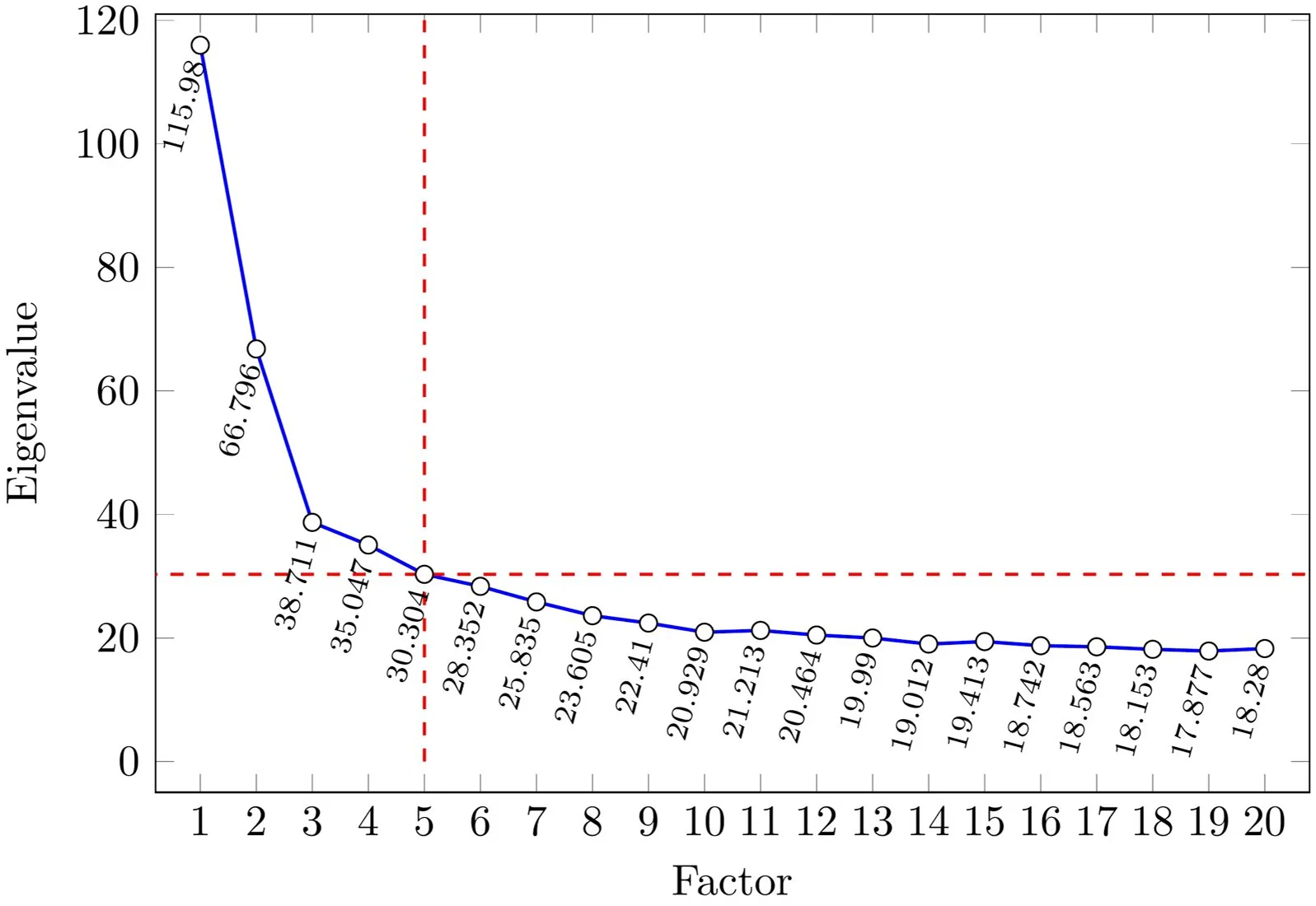
Scree plot. Eigenvalues for Factors 1–20 obtained from the lexical factor analysis. The elbow at Factor 5 supported extraction of a five-factor solution.

Each text received a score on every factor by summing the loadings of the lemmas present in that text. High-scoring texts from both poles of each factor were then analyzed to interpret the underlying discourse dimensions. Finally, analyses of variance (ANOVA) assessed the effects of persona, corpus (human vs. AI), and course on the resulting dimension scores.

## Dimensions of variation

In this section, we present the results of the Lexical Multi-Dimensional Analysis by interpreting and illustrating the discourses underlying each of the poles of the five dimensions.

### Positive dimension 1: Macro-structuring discourse.

Following standard practice in Multi-Dimensional Analysis [7,28,39], a distinction is made between primary and secondary loadings. Primary loadings are lexical items whose strongest association is with the dimension under interpretation; they are used to calculate dimension scores and guide interpretation. Secondary loadings, shown in parentheses in the factor tables, contribute to interpretation but not to scoring.

The primary loadings for positive Dimension 1 (N = 172; Table 4) characterize a discourse centered on the organization of teaching through explicit pedagogical, institutional, and conceptual references. Metadiscursive connectors such as *moreover*, *therefore*, *broadly*, and *essentially* organize the exposition, while items such as *instructional*, *framework*, *pedagogical*, *govern*, and *mandate* situate classroom activity within broader educational processes. References to *ideology*, *authority*, *power*, *reflection*, and *critical* further indicate a discourse that connects teaching practices with general principles and encourages students to examine their wider implications.

**Table 4:** Loadings for positive Dim. 1.

govern (.72), institutional (.71), instructional (.69), mandate (.66), relation (.64), national (.64), moreover (.63), network (.62), privilege (.61), intertwine (.60), researcher (.60), benefit (.57), terminology (.56), literacy (.55), imperative (.54), empower (.54), cultivate (.54), embed (.54), consciously (.53), hierarchy (.53), status (.53), integrate (.52), learner (.52), user (.51), sum (.51), rarely (.51), broadly (.50), arise (.50), instructor (.49), typically (.49), static (.48), ideological (.48), structured (.48), meaningful (.48), community (.48), robust (.47), (furthermore (.47)), narrow (.47), authority (.47), region (.47), allocate (.46), (constitute (.46)), assess (.46), contact (.46), critically (.46), difficult (.46), communicate (.46), ideology (.46), reinforce (.46), framework (.46), motivate (.45), heavily (.45), principle (.45), indigenous (.45), norm (.45), education (.45), sufficient (.44), vary (.44), solely (.44), devote (.43), united (.43), dictate (.43), measure (.43), phenomenon (.43), therefore (.42), largely (.42), focused (.42), authentic (.42), circulate (.42), broad (.42), profile (.41), multilingual (.41), basic (.41), explicitly (.41), assumption (.41), program (.41), social (.41), (thereby (.40)), interpersonal (.40), (thus (.40)), marker (.40), broaden (.40), emphasis (.40), emphasize (.40), linguistic (.40), replace (.39), essentially (.39), (inform (.39)), alongside (.39), concrete (.39), dynamics (.39), simultaneously (.38), agency (.38), secondary (.38), interestingly (.38), (boost (.38)), limited (.38), primary (.38), (additional (.38)), distinction (.38), tool (.38), critical (.38), instruction (.38), (facilitate (.37)), intersect (.37), regardless (.37), including (.37), implication (.37), (propose (.37)), actively (.37), drill (.37), pedagogical (.37), affect (.37), development (.37), individual (.37), shape (.37), context (.37), combination (.36), interplay (.36), foreground (.36), (dedicate (.36)), equally (.36), (objective (.36)), often (.36), (merely (.35)), approach (.35), practice (.35), circumstance (.34), scaffolding (.34), app (.34), repetition (.34), (attitude (.34)), (underscore (.34)), situation (.34), power (.34), reflect (.34), structure (.34), hinge (.33), (frankly (.33)), (enhance (.33)), (necessary (.33)), diverse (.33), lexical (.33), reflection (.33), (frame (.33)), study (.33), logic (.32), interface (.32), (remarkably (.32)), potential (.32), (fundamental (.32)), (regarding (.32)), address (.32), rich (.32), extremely (.31), (nowadays (.31)), overlook (.31), (viewpoint (.31)), colleague (.31), respond (.31), (similarly (.31)), tend (.31), everyday (.31), important (.31), learn (.31), (vast (.30)), (progress (.30)), exist (.30), (articulate (.30)), relate (.30), design (.30), many (.30)

This pole was therefore labeled *Macro-Structuring Discourse*. It represents teaching discourse in which immediate classroom activity is incorporated into an extended account of curricular purposes, disciplinary concepts, institutional processes, and social consequences.

Example 1, generated by GPT-4o under the talkative persona for a Language Policy class. The following excerpt illustrates its explicit organization of the lesson around broad pedagogical and sociolinguistic principles:

Text Sample 1: Positive Dim. 1: GPT-4o example

**[Speaker 1]** Before we dive into today’s text, I want to take a moment to outline why our syllabus insists on a dual focus. On one hand, we **devote** substantial time to teaching standard English. [...] On the other hand, none of you [...] live in a monolingual, monoregional bubble. [...] So our **pedagogical imperative** is twofold: ensure you can handle standard English with confidence, but also train your ear and mind to appreciate—and **critically engage with**—the full spectrum of **linguistic diversity**.

**[Speaker 1]** Bear in mind, too, that the choice of which accent or variety to **privilege** is never politically neutral. [...] It is a political stance **embedded** in your lesson plans.

The AI teacher begins by explaining the curricular purpose of the lesson and subsequently connects language teaching with linguistic diversity, professional practice, institutional power, and political decision-making. The classroom exchange is thus developed as part of a sustained disciplinary argument rather than as a series of local instructional moves.

The corresponding human text appears below.

Text Sample 2: Positive Dim. 1: Human source

**[Speaker 1]** As teachers, we should teach our students [...] what we call standard English, but we should also show them other varieties of the language. [...] Depending on the text you are translating, [...] you could have to deal with a non-standard variety.

**[Speaker 1]** When you choose as a teacher [...] to impose a specific accent, a specific variety, this is also a political decision. [...] This is food for thought. You do not have to agree.

The human teacher constructs the same discourse by relating language variation to teaching, translation, political authority, and critical reflection. GPT-4o therefore exaggerated a discourse already present in human teaching, expanding its conceptual organization and disciplinary metalanguage.

### Negative dimension 1: Performative stagecraft

The primary loadings for negative Dimension 1 (N = 71; Table 5) characterize a discourse in which classroom interaction is presented as theatrical performance. Lexical items such as *applause*, *audition*, *brace*, *riveting*, and *thrilling* evoke the language of stage performance, while evaluative terms including *masterpiece*, *pearl*, *brilliance*, and *splendid* recast routine classroom contributions as objects of artistic appraisal. The dimension also includes emotionally charged expressions, such as *torture*, *butcher*, and *existential*, producing exaggerated contrasts between praise and criticism. Together, these lexical groups define a discourse built around spectacle, dramatic evaluation, and performative interaction rather than ordinary classroom instruction.

**Table 5:** Loadings for negative Dim. 1.

dazzle (−1.00), masterpiece (−.98), riveting (−.91), brilliance (−.89), torture (−.88), feast (−.85), pearl (−.84), brace (−.80), applause (−.80), enlighten (−.77), grace (−.73), thrilling (−.73), audition (−.71), (todas (−.68)), splendid (−.68), (whining (−.67)), (su (−.67)), dusty (−.65), (momento (−.65)), brave (−.64), (este (−.63)), (ver (−.63)), (lo (−.63)), (si (−.62)), scream (−.62), (dilly (−.60)), (sin (−.59)), butcher (−.58), (dally (−.57)), existential (−.56), (clueless (−.55)), (las (−.55)), (el (−.55)), (damn (−.54)), (whine (−.53)), grand (−.52), (pathetic (−.51)), (al (−.50)), (tu (−.48)), refreshing (−.47), (decent (−.46)), (grupo (−.45)), (bien (−.45)), (los (−.44)), (delightful (−.44)), (scribble (−.43)), (ser (−.43)), (gem (−.43)), (en (−.43)), (stuff (−.42)), (rocket (−.41)), (apparently (−.41)), (sec (−.41)), (stare (−.41)), (la (−.41)), (como (−.41)), slap (−.37), awesome (−.37), soul (−.37), yep (−.36), fluff (−.36), acknowledged (−.35), (con (−.35)), lazy (−.33), symbolism (−.33), hiccup (−.33), embarrass (−.31), (es (−.31)), exciting (−.31), charming (−.31), anyway (−.31)

Example 3, generated by GPT-4o under the sarcastic persona, illustrates this discourse:

Text Sample 3: Negative Dim. 1: GPT-4o example

**[Speaker 1]** Alright, geniuses, hold your **applause** – just one more example before I unleash you into the wild...

**[Speaker 1]** So our **masterpiece**: “<name> is really keen on <name>.”

**[Speaker 1]** ...let’s move on to your next **thrilling** assignment. Oh, wait – we have yet another **riveting** example to conquer. **Brace** yourselves.

More than simply guiding students through a grammar exercise, the teacher repeatedly transforms the lesson into a staged performance. Students are positioned simultaneously as audience members and performers (“hold your applause”), while ordinary language practice is elevated through expressions such as *masterpiece*, *riveting*, and *thrilling*. The discourse continually comments on its own dramatic presentation, creating a theatrical tone that extends well beyond the instructional task itself.

The corresponding human-led class presents a markedly different interaction:

Text Sample 4: Negative Dim. 1: Human teaching

**[Speaker 1]** One more and then I’ll give you some time for you to do on your own...

**[Speaker 1]** “Keen on” is correct. But you cannot say “<name> keen on <name>.”

**[Speaker 1]** Excellent... Well done, both of you. Great. Now what I want you to do...

Here, the teacher focuses on explaining the construction, eliciting responses, and confirming correct answers. Praise remains straightforward (“Excellent,” “Well done”), and no theatrical imagery or exaggerated evaluative language is introduced. Unlike the positive pole of this dimension, where GPT-4o intensified a discourse already present in the human teaching, the present comparison shows the addition of a qualitatively different discourse. The AI overlays a theatrical, performance-oriented style onto a human classroom interaction that is procedural, supportive, and centered on the learning task.

### Positive Dimension 2: L1-scaffolded Guidance

The loadings for positive Dimension 2 (N = 32) are shown in Table 6. Unlike the other dimensions, this pole is defined almost entirely by Spanish lexical items generated for the Latin American teaching persona. Although the prompt specified only that the teacher should be Latin American, GPT-4o interpreted this as a cue to conduct substantial portions of the lesson in Spanish. While this behavior was not explicitly requested, it nevertheless reflects a plausible classroom practice, since many non-native English-speaking teachers draw on their first language to clarify concepts, organize activities, and build rapport with students.

**Table 6:** Loadings for positive Dim. 2.

momento (1.24), el (1.18), las (1.14), si (1.11), este (1.10), al (1.09), es (1.08), como (1.08), todas (1.07), ver (1.07), su (1.06), ser (1.05), tu (1.04), los (1.04), bien (1.04), grupo (1.02), con (.97), la (.96), lo (.95), por (.94), para (.94), que (.94), claro (.92), un (.85), en (.85), sin (.77), y (.75), (te (.67)), favor (.57), de (.50), ok (.48), fundamental (.46)

The lexical pattern is characterized by directive verbs (*ver*, *para*), engagement markers (*bien*, *claro*, *por favor*), collective references (*grupo*, *todas*), conditional particles (*si*, *sin*), temporal expressions, and high-frequency function words. Together, these items characterize a discourse of L1-mediated instructional scaffolding, in which the teacher organizes activities, manages transitions, and supports student participation through the learners’ shared language.

In Example 5, GPT-4o, prompted to adopt a Latin American teaching persona, conducts much of the lesson in Spanish. The teacher uses Spanish to organize the progression of the lesson, introduce new activities, explain their purpose, and maintain interaction with students.

Text Sample 5: Positive Dim. 2: AI Latin profile

**[Speaker 1]** Muy buen punto. Ahora vamos a **ver** un ejemplo concreto...

**[Speaker 1]** Recuerden **que** la mise-en-scène abarca el espacio donde ocurre la acción, el **momento** temporal y todos los objetos necesarios.

**[Speaker 1] Si** no hay preguntas, pasemos a la siguiente parte de la clase.

**[Speaker 1]**¿Les parece **si** trabajamos con fotogramas? Les propongo una actividad...

**[Speaker 1]** Por **favor**, no revelen la locación de rodaje **si** la reconocen.

**[Speaker 1]** Muy **bien**. Aquí tienen el fotograma **para que** lo analicen.

**[Speaker 1]** En grupos, extiendan ese fotograma...¿tienes alguna duda **para** comenzar?

**[Speaker 1]** Explícame **si** encuentras algún problema para iniciar la tarea.

The AI lesson is organized around procedural guidance delivered through the learners’ first language. Expressions such as *Ahora vamos a ver un ejemplo concreto*, *Si no hay preguntas*, and *Aquí tienen el fotograma para que lo analicen* introduce new activities, regulate transitions, and explain the purpose of classroom tasks. Throughout the interaction, the teacher anticipates potential difficulties, provides explicit instructions, and carefully manages the pace of the lesson.

Spanish also serves an interpersonal function. Encouragement (*Muy bien*), polite requests (*Por favor*), collaborative instructions (*En grupos*), and invitations to seek clarification (*Explícame si encuentras algún problema*) position the teacher as a supportive guide who actively monitors student participation. Rather than functioning as incidental code-switching, Spanish becomes the principal resource through which classroom management and instructional guidance are realized.

The use of Spanish by the AI teacher is not surprising in light of the prompt, which specified a Latin American persona. At the same time, the classroom context itself pointed to Portuguese: it contained references to places in Brazil, and the student turns included scattered Portuguese lexical items that GPT-4o was capable of recognizing. Nevertheless, the model privileged the statistical association between Latin American and Spanish over the immediate contextual evidence.

The human classroom interaction in Example 6 served as the basis for the AI simulation. Although the lesson is conducted entirely in English, it displays the same underlying instructional organization.

Text Sample 6: Positive Dim. 2: Human classroom

**[Speaker 1]** That’s exactly what I’m going to show you.

**[Speaker 1]** So, when you are working with films in the classroom, do you think we can work with frames?

**[Speaker 1]** If you know where this was taken, don’t say.

**[Speaker 1]** I’d like you in groups... to develop this frame into a shot.

**[Speaker 1]** Do you have any problems?

**[Speaker 1]** Let me explain. You have this frame and you have to create who she is, what she is doing...

**[Speaker 1]** Try to create the shot... I’m going to give you just five minutes.

The human teacher likewise introduces activities sequentially, organizes collaborative work, anticipates potential difficulties, and provides explicit procedural guidance. Frequent interactional markers such as “Exactly,” “Okay,” and “Well done” help maintain engagement in much the same way that *Muy bien* and *Por favor* do in the AI lesson. Likewise, conditional expressions such as “If you know where this was taken, don’t say” serve the same classroom-management function as the repeated use of *si* in the Spanish version.

Unlike the negative pole of Dimension 1, this comparison does not show GPT-4o introducing a new classroom discourse. Instead, it reproduces the instructional discourse found in the human classroom while shifting the linguistic medium from English to Spanish. The principal difference therefore lies in the systematic use of the learners’ first language as a scaffold for instruction, making a pedagogical strategy that is only implicit in the human lesson considerably more explicit in the AI-generated discourse.

### Negative dimension 2: Provocative challenge

The loadings for negative Dimension 2 (N = 74) are shown in Table 7. The lexical pattern is characterized by irony markers (*apparently*, *obvious*, *honestly*, *frankly*), confrontational verbs (*expect*, *force*, *push*, *slap*), dismissive vocabulary (*stuff*, *fluff*, *pretend*, *bother*), and conditional expressions (*unless*, *otherwise*, *never*). Together, these items characterize a discourse of provocative challenge in which classroom interaction becomes adversarial rather than collaborative. Rather than facilitating discussion, the teacher performs the role of an acerbic critic who questions students’ competence, ridicules their responses, and establishes authority through irony and confrontation.

**Table 7:** Loadings for negative Dim. 2.

(enlighten (−.76)), pretend (−.66), (damn (−.65)), (torture (−.65)), (embarrass (−.64)), slap (−.63), (brave (−.62)), stare (−.62), fluff (−.61), (pearl (−.59)), (decent (−.59)), (rocket (−.58)), apparently (−.57), (nonsense (−.56)), (scream (−.56)), bother (−.56), soul (−.55), (whine (−.53)), obvious (−.53), (dusty (−.52)), dissect (−.52), (butcher (−.49)), (lazy (−.49)), (brilliance (−.49)), (brace (−.49)), frankly (−.49), (pathetic (−.48)), (grace (−.47)), (feast (−.47)), (thrilling (−.47)), (whining (−.46)), gem (−.46), scribble (−.45), stuff (−.45), (grand (−.44)), unless (−.44), kid (−.44), (clueless (−.43)), (existential (−.43)), (dazzle (−.42)), half (−.42), (waste (−.41)), (riveting (−.40)), (splendid (−.40)), charming (−.40), excuse (−.40), throw (−.40), care (−.40), force (−.40), expect (−.40), (basically (−.39)), practically (−.39), (audition (−.37)), wrestle (−.37), nail (−.37), chatter (−.36), supposed (−.36), master (−.36), line (−.36), dear (−.35), never (−.35), honestly (−.34), sit (−.34), enough (−.34), (delightful (−.32)), otherwise (−.32), worth (−.32), pivot (−.32), (drill (−.32)), push (−.32), eye (−.32), high (−.32), stick (−.31), figure (−.30)

In Example 7, GPT-4o adopts a harsh teaching persona in a bilingualism class. The interaction is dominated by sarcasm and dismissive comments directed toward the students.

Text Sample 6: Negative Dim. 2: AI harsh style

**[Speaker 1]** All right, let’s cut the small talk. Did you watch the damn film I assigned?

**[Speaker 1]** I **expect** some substance here... or are your brains too numb?

**[Speaker 1]** <name>, **unless** you were too busy texting, tell us...

**[Speaker 1]** Look here at Portinari’s Café. What cliché emotion does this evoke?

**[Speaker 1]** Yes, sadness—**obvious**, I know. But if you were halfway competent...

**[Speaker 1] Enough** doom and gloom...

**[Speaker 1]** Of course not—if your school even **bothered** to display this mess...

**[Speaker 1]** There’s your mundane reality: assembly-**line kids** drawn onto paper like widgets.

Rather than organizing learning activities, the AI teacher organizes the interaction around confrontation. Questions such as *Did you watch the damn film I assigned?* and *What cliché emotion does this evoke?* function less as invitations to contribute than as challenges that imply inadequate student performance. Similarly, conditional expressions such as *unless you were too busy texting* presuppose student negligence before any response is given, turning ordinary requests into accusations.

Throughout the excerpt, sarcasm replaces supportive feedback. Remarks such as *or are your brains too numb?*, *halfway competent*, and *assembly-line kids drawn onto paper like widgets* ridicule students rather than encouraging reflection. Even when discussing works of art, the teacher repeatedly diverts attention toward ironic commentary and exaggerated criticism. The classroom therefore becomes a stage on which the teacher performs wit, superiority, and provocation instead of fostering collaborative inquiry.

The corresponding human classroom interaction (Example 8) presents a markedly different discourse.

Text Sample 8: Negative Dim. 2: Human classroom

**[Speaker 1]** Did you watch this film?

**[Speaker 1]** Does it change your feelings about the painting?

**[Speaker 1]** Anybody changed the feeling because it was a different way of seeing the painting?

**[Speaker 1]** The first one is Portinari. How do you feel? Tell me something.

**[Speaker 1]** This is a painting that we could use to talk to children about sadness.

**[Speaker 1]** People feel happy, you can feel anxious...

**[Speaker 1]** That’s a nice feeling about water.

**[Speaker 1]** Back at school, <name>, did you feel like this?

Like the AI teacher, the human teacher relies heavily on questions. However, these questions invite students to explore their interpretations rather than testing or ridiculing them. The discussion develops through encouragement (*That’s true*; *That’s a nice feeling*), acknowledgment of different viewpoints, and shared reflection about emotional responses to art. Questions are used to sustain dialogue, not to establish dominance.

Unlike the positive pole of Dimension 2, where GPT-4o amplifies a discourse already present in the human classroom, the negative pole introduces a qualitatively different interactional style. The human lesson contains no evidence of sarcasm, ironic performance, or adversarial authority. Instead, these features emerge almost entirely from the harsh teaching persona, transforming a collaborative classroom discussion into a confrontational performance.

### Positive dimension 3: Procedural control

The loadings for positive Dimension 3 (N = 118) are shown in Table 8. The lexical pattern is characterized by procedural verbs (*commence*, *proceed*, *ensure*, *provide*, *confirm*), analytical vocabulary (*analysis*, *inquiry*, *exploration*, *examine*), organizational terms (*outline*, *review*, *submit*, *progress*, *transition*), and politeness markers (*please*, *kindly*, *glad*). Together, these items characterize a discourse of procedural control in which the teacher explicitly organizes classroom activities, manages their progression, and supports students through structured guidance.

**Table 8:** Loadings for positive Dim. 3.

commence (.94), additionally (.73), pertinent (.72), embark (.67), kindly (.66), ensure (.63), proceed (.62), understood (.59), facilitate (.59), consult (.59), enhance (.59), delve (.56), noteworthy (.54), troubleshoot (.53), (collaboratively (.52)), outstanding (.51), remaining (.51), finding (.50), thematic (.50), conclude (.49), (furthermore (.48)), regarding (.48), outline (.48), (concur (.47)), inquiry (.46), thorough (.46), (refreshing (.46)), significantly (.46), component (.46), objective (.46), (allocate (.45)), thoughtfully (.45), analytical (.45), shall (.45), provide (.45), (thereby (.44)), intended (.44), comparative (.44), (structured (.44)), exploration (.44), (motif (.43)), (instructional (.43)), confirm (.43), analysis (.43), addition (.42), printed (.42), employ (.42), necessary (.42), engagement (.42), review (.42), please (.42), (astute (.41)), (constitute (.41)), submit (.41), progress (.41), engaging (.41), inform (.41), transition (.41), probe (.40), artistic (.40), uploaded (.40), additional (.40), (examination (.39)), retain (.39), (hiccup (.39)), dedicate (.38), propose (.38), deepen (.38), select (.38), representation (.37), selection (.37), (implication (.37)), apology (.37), annotate (.36), correspond (.36), tailor (.36), (intersect (.36)), (primary (.36)), various (.36), appropriate (.36), understanding (.36), enthusiasm (.35), indicate (.35), maintain (.35), (integrate (.35)), identify (.35), examine (.35), theoretical (.34), (comfortable (.34)), platform (.34), particularly (.34), importance (.33), strongly (.33), (including (.33)), difficulty (.33), wish (.33), compelling (.33), significant (.33), glad (.33), brief (.33), (solely (.32)), clarity (.32), enrich (.32), (address (.32)), (element (.32)), (instruction (.32)), response (.32), (acknowledged (.31)), (assess (.31)), refine (.31), viewpoint (.31), valuable (.31), (framework (.31)), clarify (.31), will (.31), (critically (.30)), straightforward (.30), require (.30)

In Example 9, GPT-4o, prompted to adopt a talkative teaching persona in a literature class, presents the lesson as a carefully organized sequence of activities. Rather than simply introducing the discussion topic, the teacher continually explains how the lesson will unfold, how students should participate, and how potential logistical issues will be handled.

Text Sample 9: Positive Dim. 3: AI talkative style

**[Speaker 1]** Today... we **will embark** on this intellectual **exploration**...

**[Speaker 1]** Let us **examine**... I **propose** we explore these media as well.

**[Speaker 1]** We **shall** form smaller colloquiums. This **will** allow for a more intricate exchange of ideas.

**[Speaker 1]** Our colloquiums are nearly ready to **proceed**. Let us **ensure** everyone finds their rightful place.

**[Speaker 1]** Before you **embark**, **kindly** document today’s questions.

**[Speaker 1]** Does anyone find themselves confused **regarding** these instructions? **Shall** I explain further?

**[Speaker 1]** Should you **require** it, I **will** gladly guide you.

**[Speaker 1]** With enthusiasm, I encourage you to **commence** your deliberations.

The AI lesson is characterized by continuous procedural management. The teacher repeatedly announces upcoming stages of the lesson, explains their purpose, confirms students’ understanding, and anticipates logistical problems before they arise. Rather than allowing activities to develop naturally, each transition is explicitly introduced and accompanied by detailed instructions.

The discourse also presents classroom interaction as an academic enterprise. Expressions such as *exploration*, *examine*, *propose*, and *intellectual* frame classroom discussion as a formal scholarly activity rather than an informal exchange of ideas. At the same time, politeness markers such as *kindly*, *gladly*, and repeated modal constructions (*shall*, *will*) soften the directive nature of the discourse. Authority is therefore exercised through institutional organization rather than through confrontation, as observed in the negative pole of Dimension 2.

The corresponding human classroom interaction (Example 10) follows the same overall lesson plan but with considerably less procedural elaboration.

Text Sample 10: Positive Dim. 3: Human classroom

**[Speaker 1]** In smaller groups we **will** be talking about these questions...

**[Speaker 1]** Let me open the groups here.

**[Speaker 1]** Some of you **will** be here with me...

**[Speaker 1]** Would you **please** take a picture of the questions?

**[Speaker 1]** Everybody understood?

**[Speaker 1]** You have to click on the button to go to your room.

**[Speaker 1]** I’m going to mute my microphone... You just keep going.

The human teacher likewise organizes breakout groups, provides instructions, and monitors participation, but does so economically. The discourse focuses on immediate classroom logistics without the extensive procedural framing found in the AI lesson. Most instructions are brief and situational, whereas GPT-4o repeatedly announces future stages, explains their rationale, and explicitly guides students through each transition.

As in Dimension 1, Dimension 3 shows the model favoring a discourse already present in the human classroom. Both teachers manage classroom procedures, but GPT-4o greatly expands the organizational component of the interaction. The result is a more formal, institutionally oriented discourse in which planning, coordination, and procedural guidance become central features of classroom talk.

### Negative dimension 3: Rapport-driven engagement

The loadings for negative Dimension 3 (N = 66) are shown in Table 9. Although relatively few lexical items load primarily on this pole, they consistently signal conversational involvement. Primary loadings include affective expressions (*funny*, *laugh*), informal references (*guy*, *somebody*), evaluative language (*lazy*, *anymore*), and the Spanish clitic pronoun *te*. Together, these items characterize a discourse of rapport-driven engagement in which instruction is embedded within informal conversation, humor, and personal interaction.

**Table 9:** Loadings for negative Dim. 3.

te (−.99), (las (−.74)), (su (−.74)), (es (−.72)), (los (−.71)), (si (−.69)), (este (−.68)), (tu (−.68)), (el (−.68)), (lo (−.68)), (al (−.67)), (como (−.64)), (todas (−.63)), (momento (−.63)), (grupo (−.62)), (embarrass (−.61)), (con (−.61)), (ver (−.60)), (y (−.60)), (bien (−.59)), (sin (−.57)), (claro (−.57)), (pathetic (−.55)), (ser (−.54)), (que (−.54)), lazy (−.53), hover (−.52), (slap (−.52)), (yeah (−.52)), somebody (−.50), funny (−.49), (fluff (−.47)), (stare (−.46)), anymore (−.45), (damn (−.44)), (amazing (−.42)), (la (−.42)), (por (−.42)), (audition (−.41)), laugh (−.41), (rocket (−.40)), (um (−.38)), (whining (−.37)), (butcher (−.37)), (anybody (−.37)), (un (−.37)), (decent (−.36)), (stuff (−.36)), (soul (−.36)), (wow (−.36)), (whine (−.35)), (super (−.34)), guy (−.34), (yep (−.33)), (dusty (−.33)), (torture (−.32)), (chatter (−.32)), (supposed (−.32)), (nowadays (−.32)), (hey (−.32)), (pearl (−.31)), (apparently (−.31)), (anyway (−.31)), (totally (−.31)), (clueless (−.30)), (brace (−.30))

Most of the remaining lexical items are secondary loadings, many of them Spanish function words associated primarily with positive Dimension 2. Their presence reflects overlap between bilingual classroom interaction and informal interpersonal engagement, but they do not define this dimension. The primary discourse is instead organized around conversational exchanges that reduce social distance between teacher and students.

Example 11 illustrates this discourse. GPT-4o, prompted to adopt the Latin American teaching persona in a literature class, develops the discussion through humor, informal dialogue, and occasional Spanish code-switching.

Text Sample 11: Negative Dim. 3: AI Latin style

**[Speaker 19]** I think it’s a little bit **funnier** than the first one.

**[Speaker 1]** I see your point that the second scene makes us **laugh** more... Sometimes what we find **funny** is actually our discomfort filling in the blanks. What do you all think?

**[Speaker 1]** <name>,¿y tú qué piensas de esto?

**[Speaker 1]**¿Alguien más quiere comentar...?¿Qué **te** pareció a ti?

**[Speaker 1]** ¡Muy bien! Esa es una observación aguda...

**[Speaker 1]** Recuerden que Brioni estaba viendo sólo imágenes...

The AI teacher develops the discussion conversationally instead of through formal explanation. The student’s characterization of the scene as ‘funnier’ becomes the starting point for a collaborative exploration of interpretation. Humor is treated as a legitimate interpretive response, and the teacher repeatedly invites alternative viewpoints through open questions such as *What do you all think?*

The discourse is personalized through code-switching. Spanish is used selectively to address individual students (*¿Qué te pareció?*), provide encouragement (*¡Muy bien!*), and maintain the flow of interaction. Unlike positive Dimension 2, where Spanish primarily serves instructional scaffolding, here it functions as an interpersonal resource that reinforces familiarity and shared participation. Throughout the excerpt, the teacher adopts the stance of a discussion partner rather than a formal instructor.

The corresponding human classroom interaction (Example 12) displays the same conversational orientation, although less strongly.

Text Sample 12: Negative Dim. 3: Human classroom

**[Speaker 19]** I think it’s a little bit more **funnier** than the first one.

**[Speaker 1]** I’m not so sure if I agree with this **funnier**, but okay.

**[Speaker 1]** Other comments?

**[Speaker 1]** Exactly. Okay. Well done.

**[Speaker 1]** Remember that Brioni... she is kind of inventing things.

**[Speaker 1]** That’s what Brioni is doing.

The human teacher likewise maintains an informal discussion in which students’ interpretations drive the lesson. The teacher accepts the student’s characterization of the scene as ‘funnier’ and responds conversationally (*I’m not so sure if I agree... but okay*) before encouraging additional contributions. Frequent acknowledgments (*Exactly*, *Well done*) sustain the interaction while allowing students’ ideas to remain central to the discussion.

Unlike the previous dimensions, the difference between GPT-4o and the human classroom is relatively small. Both organize the lesson around conversational participation rather than formal exposition. GPT-4o primarily amplifies this discourse by making greater use of humor, interpersonal comments, and Spanish expressions that reinforce rapport, but the underlying interactional style is already present in the human classroom.

### Positive dimension 4: Evaluative control

The loadings for positive Dimension 4 (N = 46) are shown in Table 10. The lexical pattern is characterized by harsh evaluative language (*pathetic*, *clueless*, *nonsense*, *whine*, *embarrass*), expressions that regulate classroom pace (*dilly*, *dally*, *waste*), and restrained approval (*satisfactory*, *decent*, *acknowledged*, *yep*). Together, these items characterize a discourse of evaluative control in which the teacher manages classroom interaction through continual judgment of student performance rather than through explanation or encouragement.

**Table 10:** Loadings for positive Dim. 4.

dilly (.97), whining (.92), pathetic (.89), dally (.84), embarrass (.82), acknowledged (.81), whine (.81), damn (.77), yep (.77), satisfactory (.75), decent (.75), clueless (.69), rocket (.68), (grupo (.66)), (todas (.65)), nonsense (.63), (momento (.63)), (ser (.63)), collaboratively (.61), (understood (.55)), waste (.54), (por (.54)), (este (.52)), (tu (.51)), (butcher (.49)), (awesome (.49)), (si (.48)), (el (.47)), (al (.46)), (ver (.46)), (troubleshoot (.45)), (instructional (.44)), (las (.42)), chalkboard (.41), (lazy (.41)), (anybody (.41)), (su (.40)), (es (.40)), (ok (.39)), (yeah (.39)), (super (.35)), (lo (.35)), (claro (.34)), (fix (.34)), (audition (.32)), (allocate (.32))

In Example 13, GPT-4o, prompted to adopt the harsh teaching persona in a C1 English class, continually evaluates students’ answers while maintaining tight control over the pace of the lesson.

Text Sample 13: Positive Dim. 4: AI harsh style

**[Speaker 1]** Rice—explain yourself.

**[Speaker 1]** Cooking rice is embarrassingly simple.

**[Speaker 1]** Thanks, I guess that satisfied your ego.

**[Speaker 1]** “My schoolwork”—**pathetic**, I know.

**[Speaker 1]** I used to get pats on the head for **decent** schoolwork.

**[Speaker 1]** Now stop **whining**, pair up..

**[Speaker 1]** No **whining**

**[Speaker 1]** Use the **damn** prepositions... Any last **pathetic** questions?

Throughout the excerpt, the teacher evaluates virtually every student contribution. The teacher judges, dismisses, or ridicules students’ answers instead of acknowledging and building on them. Expressions such as *pathetic*, *damn*, and repeated references to *whining* create a classroom in which students’ ideas become objects of evaluation rather than opportunities for discussion.

Even positive feedback is deliberately restrained. The adjective *decent* provides only minimal approval, while remarks such as *Thanks, I guess that satisfied your ego* immediately undermine any apparent acknowledgment. Praise therefore functions less as encouragement than as another mechanism for maintaining authority.

Frequent imperatives (*Explain yourself*; *Pair up*; *Use the prepositions*) accelerate the lesson while limiting opportunities for negotiation or reflection. Classroom management becomes a vehicle for continual performance assessment rather than for supporting learning.

The corresponding human classroom interaction (Example 14; score = 0) follows the same instructional sequence without introducing evaluative pressure.

Text Sample 14: Positive Dim. 4: Human classroom

**[Speaker 1]** Rice. Why?

**[Speaker 1]** Thank you. Next one...

**[Speaker 1]** Why did I write a laptop and headphones?

**[Speaker 1]** I used to be congratulated on my schoolwork.

**[Speaker 1]** Now with your neighbors you’re going to do the same thing.

**[Speaker 1]** Any questions before we go?

The human teacher likewise guides the activity, asks students to justify their answers, and organizes pair work, but evaluation remains neutral. Questions are used to elicit responses rather than expose weaknesses, and instructions are delivered without ridicule or sarcasm. Although the teacher directs the lesson, authority is exercised through ordinary classroom management rather than through continual judgment of student performance.

Like the negative pole of Dimension 2, this dimension illustrates how GPT-4o introduces a discourse largely absent from the human classroom. The instructional sequence remains essentially the same, but the AI transforms it into a performance in which criticism, restrained praise, and continual evaluation become the dominant means of regulating classroom interaction.

### Negative dimension 4: Reflective aesthetic engagement

The loadings for negative Dimension 4 (N = 118) are shown in Table 11. The lexical pattern is characterized by aesthetic vocabulary (*tapestry*, *motif*, *landscape*, *frame*, *visual*), emotionally charged language (*heart*, *spirit*, *emotional*, *resonate*, *mood*), and interpretive verbs (*reveal*, *underscore*, *illustrate*, *capture*, *reframe*, *weave*). Together, these items characterize a discourse of reflective aesthetic engagement in which language, literature, and culture are interpreted through symbolic, emotional, and artistic perspectives rather than treated primarily as objects of technical analysis.

**Table 11:** Loadings for negative Dim. 4.

tapestry (−.60), profound (−.59), raw (−.58), shade (−.57), delightful (−.53), living (−.53), tradition (−.49), rhythm (−.49), emotional (−.48), alter (−.47), gentle (−.46), striking (−.46), beautifully (−.46), intense (−.45), spirit (−.45), heart (−.45), resonate (−.45), embrace (−.44), intriguing (−.44), (soul (−.43)), heighten (−.43), motif (−.43), charge (−.43), connotation (−.43), lens (−.43), reflective (−.42), shine (−.42), presence (−.42), deliberate (−.42), color (−.42), powerful (−.42), emotionally (−.41), (grand (−.41)), symbolism (−.41), symbolize (−.41), cast (−.41), self (−.41), merely (−.40), weave (−.40), underscore (−.40), journey (−.40), fascinating (−.40), wave (−.39), (artistic (−.39)), brilliant (−.39), playful (−.39), landscape (−.39), spark (−.39), wonderfully (−.38), threat (−.38), interrogate (−.38), filmmaker (−.38), mid (−.38), attitude (−.38), (exploration (−.38)), (delve (−.38)), preserve (−.38), (existential (−.37)), gathering (−.37), celebrate (−.37), lovely (−.37), surface (−.37), element (−.37), capture (−.37), musical (−.36), absorb (−.36), (agency (−.36)), atmosphere (−.36), storytelling (−.36), (astute (−.35)), (la (−.35)), remarkably (−.35), honor (−.35), mood (−.35), curiosity (−.35), era (−.35), similarly (−.35), reveal (−.35), tone (−.35), almost (−.35), firmly (−.34), (retain (−.34)), land (−.34), mirror (−.34), (shape (−.34)), (interplay (−.33)), shifting (−.33), vast (−.33), harsh (−.33), prime (−.33), (communicate (−.33)), boundary (−.33), classic (−.33), visual (−.33), frame (−.33), perhaps (−.33), illustrate (−.33), carry (−.33), (laugh (−.32)), (compelling (−.32)), path (−.32), (lexical (−.32)), (emphasize (−.32)), insightful (−.32), reframe (−.31), recognition (−.31), uncover (−.31), eager (−.31), (thematic (−.31)), (employ (−.31)), (dear (−.31)), articulate (−.31), shift (−.31), (exciting (−.30)), thread (−.30), (everyday (−.30)), (broad (−.30)), narrative (−.30)

In Example 15, GPT-4o, prompted to adopt the critical teaching persona in a Language Awareness class, repeatedly interprets linguistic phenomena through aesthetic and symbolic language.

Text Sample 15: Negative Dim. 4: AI critical style

**[Speaker 1]** Recife... **celebrated** for its mangue **rhythms**...

**[Speaker 1]** That sounds **delightful**.

**[Speaker 1]** Language doesn’t **merely** label reality; it actively constructs it.

**[Speaker 1]** I’d like to focus on terms that **reveal** power dynamics...

**[Speaker 1]** Ramos **underscores** how vocabulary becomes a marker of social exclusion.

**[Speaker 1]** This entire excerpt **resonates** with our theme.

**[Speaker 1]** Words **carry** different registers and **connotations**.

**[Speaker 1]** These words signal different **shades** of meaning.

**[Speaker 1]** Choosing between them positions speakers within a social **landscape**.

The AI teacher interprets what vocabulary signifies instead of focusing primarily on the vocabulary itself. Lexical choices become evidence of broader cultural, social, and emotional processes. Literature is discussed not simply as a source of examples but as a vehicle through which questions of identity, exclusion, and meaning are explored.

Throughout the lesson, interpretation is supported by metaphorical language. Expressions such as *social landscape*, *carry different registers*, *resonates with our theme*, and *reveal power dynamics* invite students to understand linguistic choices as symbolic rather than merely referential. Emotional vocabulary likewise becomes part of the analytical process, encouraging students to consider not only what language denotes but also how it evokes attitudes, values, and social relationships.

The corresponding human classroom interaction (Example 16) addresses essentially the same linguistic content while adopting a substantially different discourse.

Text Sample 16: Negative Dim. 4: Human classroom

**[Speaker 1]** If he knew how to speak, he would argue...

**[Speaker 1]** The parrot didn’t speak...

**[Speaker 1]** Lack of words... lack of vocabulary...

**[Speaker 1]** We have words from Old English, French, Latin and Greek.

**[Speaker 1]** Ask, question, **interrogate**.

**[Speaker 1]** Scare, frighten, terrify.

**[Speaker 1]** Kind, generous, magnanimous.

**[Speaker 1]** English has specific words for every situation.

Although the human teacher likewise examines lexical meaning and discusses the literary text *Vidas Secas*, the discussion is organized primarily through comparison, exemplification, and classification. Word sets such as *ask–question–interrogate* and *scare–frighten–terrify* illustrate semantic and etymological distinctions directly, with relatively little metaphorical or emotional elaboration. Literary examples support linguistic explanation rather than becoming objects of extended symbolic interpretation.

Unlike the positive pole of Dimension 4, where GPT-4o introduces evaluative pressure absent from the human classroom, the negative pole represents a stronger manifestation of an existing human discourse. Both teachers interpret literature and lexical meaning, but GPT-4o devotes substantially more attention to their symbolic, aesthetic, and affective dimensions. As a result, interpretation occupies a larger portion of the interaction and more frequently invokes metaphor, symbolism, and emotional meaning than in the corresponding human classroom discourse.

### Positive dimension 5: Informal facilitation

The loadings for positive Dimension 5 (N = 114) are shown in Table 12. The lexical pattern is characterized by conversational markers (*uh*, *um*, *oh*, *hey*, *hi*), positive evaluations (*awesome*, *amazing*, *great*, *wonderful*, *fun*), engagement markers (*everyone*, *everybody*, *okay*, *thanks*), technology-related vocabulary (*Zoom*, *slide*, *screen*, *check*, *site*), and hedging expressions (*maybe*, *pretty*, *just*, *right*, *sure*). Together, these items characterize a discourse of informal facilitation in which the teacher manages classroom activities through conversational interaction rather than institutional formality.

**Table 12:** Loadings for positive Dim. 5.

super (.86), uh (.73), lots (.69), awesome (.68), hey (.68), totally (.66), thumb (.65), sec (.64), hi (.59), plenty (.58), yeah (.58), amazing (.57), pretty (.56), anybody (.55), handy (.55), hmm (.54), (yep (.53)), everybody (.52), huge (.52), okay (.51), exciting (.49), hop (.49), somewhere (.49), tiny (.49), tip (.49), definitely (.49), sorry (.49), (troubleshoot (.48)), wow (.48), fix (.48), zoom (.48), fun (.48), quick (.48), hello (.47), usually (.47), crystal (.46), basically (.46), hang (.46), (ok (.45)), folk (.45), fantastic (.45), oh (.45), hiccup (.44), um (.44), nice (.44), tweak (.43), excited (.43), tough (.43), boost (.42), grab (.42), yesterday (.41), tech (.41), everyone (.41), incredible (.40), scan (.40), energy (.40), couple (.40), check (.40), worry (.40), (stuff (.39)), chunk (.39), flip (.39), able (.39), get (.39), crowd (.38), honest (.38), (throw (.38)), lot (.38), jot (.38), maybe (.38), bit (.38), thanks (.38), nowadays (.37), shout (.37), smooth (.37), comfortable (.37), great (.37), go (.37), working (.36), nicely (.36), alright (.36), launch (.35), anyway (.35), kick (.35), main (.35), thing (.35), really (.35), flag (.34), generally (.34), pull (.34), job (.34), confidently (.33), (app (.33)), site (.33), slide (.33), just (.33), setup (.32), tune (.32), virtual (.32), hope (.32), big (.32), try (.32), right (.32), (wonderfully (.31)), (funny (.31)), (laugh (.31)), (glad (.31)), walk (.31), cool (.30), disappear (.30), helpful (.30), wonderful (.30), stay (.30), sure (.30)

In Example 17, GPT-4o, prompted to adopt the anxious teaching persona in a bilingualism class, organizes the lesson through a highly conversational style. The teacher sounds anxious by constantly checking in, reassuring students, softening directives, hedging, apologizing, and revising plans.

Text Sample 17: Positive Dim. 5: AI anxious style

**[Speaker 1]** H-hello **everyone**. **Uh**, I **hope** you’re all doing **okay** today...

**[Speaker 1] Great**. The plan is...

**[Speaker 1] Okay**, I’m going to open the breakout rooms now.

**[Speaker 1] Hi**! **Quick check**: did **Zoom** assign you to a room?

**[Speaker 1]** Hang on **just** a **sec**...

**[Speaker 1]** No **worries**.

**[Speaker 1] Maybe** it’s simpler if we **just stay** in the main room.

**[Speaker 1]** Feel free to disagree—that’s the **fun** part.

**[Speaker 1]** I’ll **hop** around other groups...

The anxiety reduces the formality of classroom management. Transitions are introduced conversationally through greetings, apologies, reassurance, and spontaneous comments instead of concise instructions. Frequent fillers, hedges, and self-repairs create the impression of an interaction that develops collaboratively rather than according to a fixed script.

The teacher also treats technological issues as part of the ordinary conversation. References to Zoom, breakout rooms, slides, screenshots, and internet connections are integrated naturally into the interaction, while reassuring expressions such as *No worries* and *Hang on just a sec* help maintain participation despite logistical interruptions. Throughout the lesson, procedural management is embedded in informal dialog.

The corresponding human classroom interaction (Example 18) displays the same discourse.

Text Sample 18: Positive Dim. 5: Human classroom

**[Speaker 1]** Is it **okay**?

**[Speaker 1] Hi**, <name>.

**[Speaker 1]** Let me see if I can put you in a room, **just** a second.

**[Speaker 1] Sure**, I can explain.

**[Speaker 1]** That’s **okay**.

**[Speaker 1]** I’m going to go to the other rooms.

**[Speaker 1]** Did you have time to see the news a **bit**?

**[Speaker 1]** Focus on the picture... the **slide** that is **big**.

Like the AI teacher, the human teacher manages the lesson conversationally. Greetings, reassurance, brief explanations, and references to the online platform are woven naturally into classroom management. Instructions emerge through ordinary dialogue instead of through procedural language, thereby creating an approachable and collaborative atmosphere.

Among all five dimensions, this is the one in which GPT-4o most faithfully reproduces the discourse of the human classroom. The AI does not introduce a qualitatively different discourse. Fillers, positive evaluations, informal transitions, and engagement markers are frequent, producing a more pronounced version of an already highly informal and facilitative discourse.

### Negative dimension 5: Formal deductive exposition

The loadings for negative Dimension 5 (N = 53) are shown in Table 13. The lexical pattern is characterized by logical connectives (*thus*, *thereby*, *furthermore*), evaluative metadiscourse (*concur*, *astute*), and abstract conceptual vocabulary (*constitute*, *examination*, *evolution*). Together, these items characterize a discourse of formal deductive exposition in which classroom interaction is organized around systematic explanation, conceptual development, and explicit logical progression rather than conversational engagement.

**Table 13:** Loadings for negative Dim. 5.

concur (−.99), (todas (−.84)), thereby (−.77), (acknowledged (−.76)), (satisfactory (−.74)), (los (−.73)), (pertinent (−.71)), (claro (−.70)), (las (−.66)), (momento (−.65)), (tu (−.60)), (al (−.60)), thus (−.60), (este (−.59)), (el (−.59)), (ver (−.56)), astute (−.56), (si (−.55)), furthermore (−.54), (commence (−.53)), (grupo (−.52)), (lo (−.52)), (ser (−.51)), (noteworthy (−.51)), constitute (−.51), (imperative (−.47)), (te (−.47)), (la (−.45)), (sin (−.44)), (additionally (−.44)), (damn (−.42)), (su (−.42)), examination (−.41), (clueless (−.40)), (pearl (−.40)), (pathetic (−.38)), (embarrass (−.37)), (heighten (−.37)), (y (−.37)), (dally (−.36)), (allocate (−.36)), (intertwine (−.36)), (facilitate (−.36)), (govern (−.35)), (dilly (−.33)), (instructional (−.33)), (como (−.32)), (profound (−.32)), (moreover (−.32)), evolution (−.31), (dictate (−.31)), (merely (−.31)), (whine (−.30))

Example 19 illustrates this discourse. GPT-4o, prompted to adopt the strict teaching persona in a Language Policy class (score = −4), responds briefly to a student’s comment before developing an extended conceptual explanation of Halliday’s metafunctions.

Text Sample 19: Negative Dim. 5: AI strict style

**[Speaker 1]** That is an **astute** observation...

**[Speaker 1]** We will now introduce Halliday’s central proposition...

**[Speaker 1]** This representational aspect **constitutes** Halliday’s first metafunction.

**[Speaker 1] Furthermore**, within this function...

**[Speaker 1] Thus**, the ideational function...

**[Speaker 1]** The second metafunction..

**[Speaker 1]** Finally, the third metafunction...

Once the student contribution has been acknowledged, the discourse becomes largely expository. The teacher develops a sequence of propositions that progressively construct the theoretical framework instead of sustaining dialog. Each concept follows logically from the previous one through explicit connectives such as *furthermore* and *thus*, producing an organized deductive presentation.

The teacher also adopts an authoritative epistemic stance. Student contributions are evaluated through expressions such as *That is an astute observation*, while concepts are presented as settled theoretical knowledge rather than topics for collaborative exploration. Abstract nominalizations and formal vocabulary reinforce the impression of academic exposition, giving the interaction the character of a university lecture.

The corresponding human classroom interaction (Example 20) covers essentially the same theoretical material but organizes it differently.

Text Sample 20: Negative Dim. 5: Human classroom

**[Speaker 1]** Yes, good. I love Mia Couto.

**[Speaker 1]** We are not going to finish this tonight.

**[Speaker 1]** According to Halliday...

**[Speaker 1]** The first function...

**[Speaker 1]** The second one...

**[Speaker 1]** And the last function...

Like the AI teacher, the human teacher presents Halliday’s metafunctions sequentially. However, the explanation is punctuated by interactional markers such as *Yes?*, *OK?*, and *Right?*, together with comments addressed directly to the students. These conversational interruptions maintain audience involvement and prevent the discourse from becoming a continuous lecture.

Both teachers explain the same theoretical framework, but GPT-4o presents it as a formal exposition built through a sequence of logically connected propositions. Explicit logical progression, conceptual precision, and sustained exposition replace the more conversational development found in the human classroom. As before, the model thus gives greater prominence to an instructional discourse already present in the human classroom while preserving the same pedagogical style.

## Analysis of variance

To assess the extent to which teaching personas differ in their use of the five discourse dimensions, a one-way analysis of variance (ANOVA) was performed for each dimension, using persona as the grouping factor. Fig 2 summarizes the distributions of dimension scores for all personas, while Table 14 reports the corresponding omnibus ANOVA statistics.

**Fig 2:**
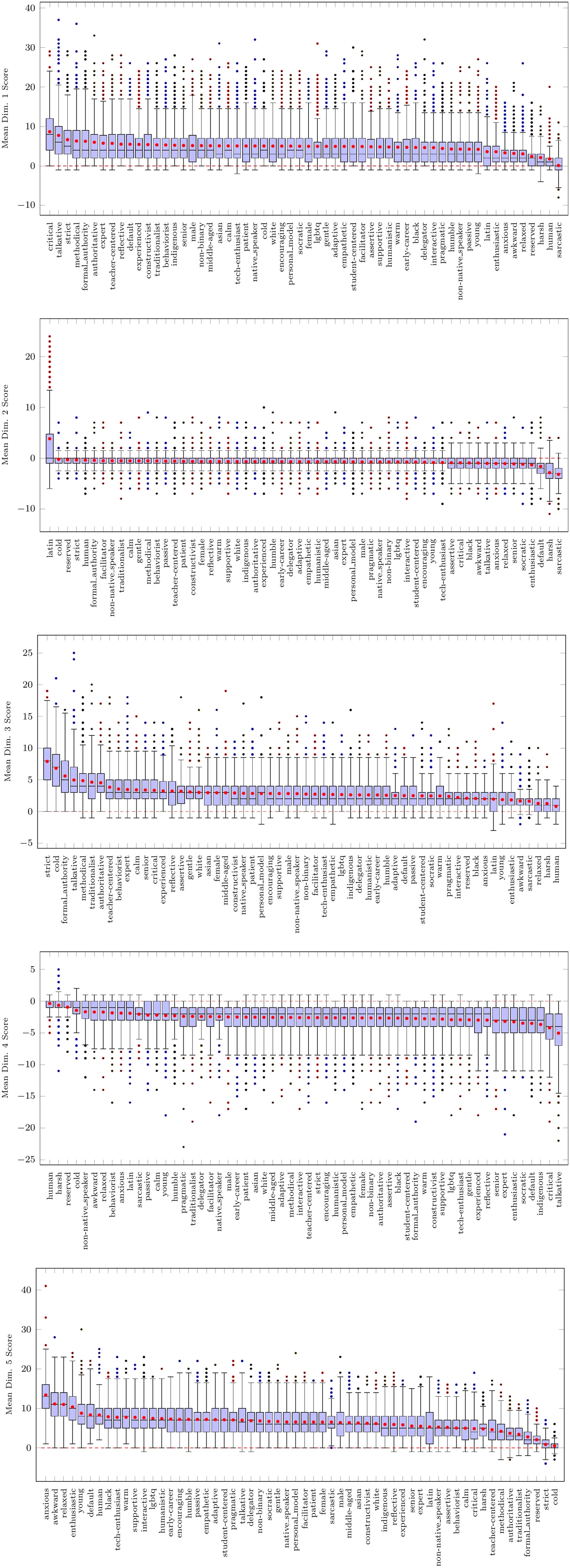
Boxplots of dimension scores by teaching persona. Vertical mosaic showing the distributions of dimension scores for the teaching personas on Dimensions 1–5. Each panel presents the median (horizontal line), interquartile range (box), whiskers, outliers, and mean score (filled circle), allowing comparison of the central tendency and variability of the persona distributions across the five dimensions.

**Table 14:** One-way ANOVA results for the effect of teaching persona on dimension scores.

Dimension	*F*	*p*	*R* ^2^	Variance explained (%)
1	25.67	< .0001	0.067	6.7
2	74.10	< .0001	0.173	17.3
3	99.59	< .0001	0.219	21.9
4	29.83	< .0001	0.078	7.8
5	130.56	< .0001	0.269	26.9

All five analyses were statistically significant (*p* < .0001), indicating that the personas differ systematically in the degree to which they realize each discourse dimension. The magnitude of these differences, however, varied across the dimensions. Dimension 5 exhibited the strongest differentiation among personas, accounting for 26.9% of the variance in dimension scores, followed by Dimension 3 (21.9%) and Dimension 2 (17.3%). By contrast, Dimensions 1 and 4 showed modest effects, explaining only 6.7% and 7.8% of the variance, respectively.

Fig 2 shows the distribution of persona scores for each dimension. Only Dimensions 3 and 5 exhibit substantial separation among the personas, with the highest- and lowest-scoring personas clearly distinguished from the remainder. With Dimension 2, the differentiation is driven primarily by a single outlying persona, while most of the remaining personas are compressed into a narrow range of scores. The same flattening of the middle-ranked personas is also evident in the other dimensions, where much of the discrimination occurs at the extremes of the continuum.

## Discussion

In this section, we summarize the findings, relate them to previous literature, and answer the research questions.

### RQ1. What are the major discourse dimensions through which GPT-4o constructs the teacher role?

This study identified five dimensions comprising ten complementary discourses. Together, these dimensions constitute a model of AI-generated teaching discourse. Broadly, the ten dimension poles reflect three major domains of pedagogical discourse. First, instructional discourse – this group encompasses the organization and delivery of instruction (positive Dimension 1, positive Dimension 3, and negative Dimension 5). Second, interactional discourse, which concerns the teacher’s interpersonal positioning toward students, ranging from guidance and rapport to confrontation and evaluation (positive and negative Dimensions 2 and 4, together with negative Dimension 3 and positive Dimension 5). And thirdly, performative discourse, a specialized discourse which captures the enactment of the teaching role itself through theatricality and staged classroom performance (negative Dimension 1).

### RQ2. How are AI-generated teaching discourses positioned relative to human classroom discourse?

Comparison with the corresponding human classroom teaching revealed two principal patterns. First, most AI discourses represented amplifications of discourse already present in the human classroom, such as Macro-Structuring (positive Dimension 1), L1-scaffolded Guidance (positive Dimension 2) and Procedural Control (positive Dimension 3). In these cases, GPT-4o reproduced the underlying discourse while making it more explicit, coherent, or stylistically marked.

Second, three discourses appeared as novel LLM discourses: Performative Stagecraft (negative Dimension 1), Provocative Challenge (negative Dimension 2), and Evaluative Control (positive Dimension 4). This suggests that an LLM can introduce artificial styles that are uncommon in naturally occurring classroom interaction.

The ability for LLMs to display a ‘cleaned up,’ hyper-articulate stylization of human interaction has been observed in the literature. For instance, the pedagogical amplification where GPT-4o heightened structural, scaffolding, and relational moves reflects the literature on LLM capabilities in education, which highlights the models’ capacity to generate highly organized instructional routines compared to spontaneous human talk [40,41]. This tendency for LLMs to strip away the ‘messiness’ of human speech is noted by [42], who note that LLM discourse feels pristine and overly scripted. Also, Berber Sardinha (2004) demonstrated that LLMs struggle to capture the spontaneous, fragmented nature of natural human talk, defaulting instead to hyper-standardized grammatical structures and explicit rhetorical markers [43].

To provide a statistical comparison between the overall GPT-generated discourses and the human teaching discourse, we fit a series of linear mixed-effects models with discourse dimension score as the dependent variable, group (Human vs. AI) as a fixed effect, and source human text as a random intercept. As shown in Table 15, GPT-generated texts differed significantly from the human texts on all five discourse dimensions (all *p* < .001). GPT-generated texts scored significantly higher on the positive pole of Dimensions 1 (Macro-Structuring) and 3 (Procedural Control), and significantly higher on the negative pole of Dimensions 2 (Provocative Challenge), 4 (Reflective Aesthetic Engagement), and 5 (Formal Deductive Exposition).

**Table 15:** Mixed-effects model comparisons between human and AI-generated texts across the five discourse dimensions. Human and AI columns report the estimated marginal means from the mixed-effects models. The Estimate column reports the difference between the estimated marginal means (AI-Human).

Dimension	Poles	Human	AI	Estimate	*F*	*p*
1	Macro-Structuring vs. Performative Stagecraft	1.77	4.86	3.09	486.1	< .001
2	L1-scaffolded Guidance vs. Provocative Challenge	−0.39	−0.73	−.34	14.8	< .001
3	Procedural Control vs. Rapport-driven Engagement	0.81	3.01	2.19	381.6	< .001
4	Evaluative Control vs. Reflective Aesthetic Engagement	−0.36	−2.58	−2.22	587.0	< .001
5	Informal Facilitation vs. Formal Deductive Exposition	8.31	6.39	−1.91	120.9	< .001

### RQ3. To what extent do teacher personas differ in their positions on the discourse dimensions identified by LMDA?

The teacher personas examined in this study represent a range of pedagogical styles, professional identities, interpersonal orientations, and instructional roles. These distinctions would be expected to give rise to different forms of teaching discourse. Previous research shows distinct teaching styles, personalities, and identities differ in their instructional and interpersonal practices [8,11,14,15,19,20]. The present findings, however, show only limited differentiation across the personas. Although some personas favored particular discourses, most occupied similar positions on the five dimensions, with substantial overlap. Thus, GPT-4o realized a diverse set of persona specifications through a comparatively restricted repertoire of pedagogical discourses that was only weakly differentiated by persona.

To provide a statistical assessment of the extent to which the teacher personas differed from one another, we conducted all pairwise comparisons among the 57 AI personas for each discourse dimension using Holm-adjusted estimated marginal means derived from the mixed-effects models. As shown in Table 16, the degree of differentiation varied considerably across the five dimensions. Dimension 2 exhibited the greatest convergence, with 75.3% of the 1,596 pairwise comparisons being non-significant after Holm correction, indicating that most personas occupied statistically indistinguishable positions along this dimension. By contrast, Dimension 5 showed the greatest differentiation, with only 25.4% of comparisons remaining non-significant. Dimensions 1 and 4 occupied an intermediate position, with approximately half of the persona pairs differing significantly, whereas Dimension 3 showed somewhat greater differentiation.

**Table 16:** Differentiation among AI personas along the five discourse dimensions based on all pairwise comparisons (57 personas; 1,596 comparisons per dimension).

Dimension	Significant	Non-significant	Non-significant (%)
1	836	760	47.6
2	394	1202	75.3
3	988	608	38.1
4	829	767	48.1
5	1191	405	25.4

### Implications

The implications of our study are threefold. The first one concerns the use of persona prompting in AI-assisted education. If an educational application requires an LLM to adopt a particular teaching persona, the present results suggest that GPT-4o is unlikely to realize that persona through a uniquely identifiable discourse. Although different personas produced statistically distinguishable discourse in some dimensions, most occupied overlapping regions of the discourse space. Consequently, new personas are therefore more likely to instantiate different combinations of the discourses already identified than to reveal actual new teaching discourses.

A second implication concerns research on AI-generated teaching discourse. The five discourse dimensions identified in this study constitute a discourse model of AI teaching voices. Because the model was induced from a corpus representing more than fifty teacher personas, it provides a stable framework for describing and comparing AI-generated teaching discourse. Future studies may therefore use the model to examine other LLMs, prompt designs, educational domains, or teaching tasks within a common descriptive framework.

A final implication is that the findings suggest that the LLM possesses multiple teaching voices rather than a single, uniform instructional style. However, the LLM has multiple teaching voices, but not an open-ended repertoire of individual voices. That is, these voices do not correspond to an equally rich set of individual pedagogical identities. Instead, the model appears to realize many distinct persona specifications through a comparatively limited repertoire of discourse configurations. In this sense, GPT-4o exhibits variation in teaching discourse, but the range of that variation remains constrained, limiting the extent to which persona prompting alone can generate genuinely distinctive pedagogical voices.

## Conclusion

This study examined how GPT-4o and human teachers realize teaching through discourse by applying Lexical Multi-Dimensional Analysis to a corpus of simulated and authentic classroom interactions. It introduced a five-dimensional model of teaching discourse comprising ten discourse poles and used it to compare the discursive organization of AI- and human-generated teaching.

The findings show that GPT-4o can produce some coherent and well-organized instructional language. At the same time, the model frequently exaggerated a few persona-defining traits, resulting in artificial pedagogical voices. More generally, while a few personas were exaggerated, most converged on largely undifferentiated pedagogical discourses. Thus, GPT-4o realized a diverse set of teacher personas through comparatively limited discursive variation.

The limitations in the current study include the fact that it examined only one language model, relied on simulated rather than interactive classroom communication, and did not assess pedagogical effectiveness. Because the corpus was generated exclusively with GPT-4o (an advanced model at the time, March 2025), the discourse dimensions identified here should be interpreted as characterizing GPT-4o under the prompting protocol adopted in this study. Whether the same discourse organization, persona clustering, or hybridization patterns emerge in other LLMs remains an empirical question requiring comparative investigation. Future research should extend the analysis to other LLMs, investigate multidimensional discourse configurations across personas, compare AI discourse with educational proficiency benchmarks, and evaluate AI-generated teaching in authentic classroom settings.

## Supporting information

S1 Appendix Teacher personas. (PDF)
