# Supplementary material for: When AI simulates teaching personas: Linguistic dimensions of LLM-generated classroom discourse
Source: PLoS One. 2026 Nov 6;21(11):e0360650. doi: 10.1371/journal.pone.0360650 (PMC13742886; doi:10.1371/journal.pone.0360650)
Supplement: Supplementary file 1 [file pone.0360650.s001.pdf]

Supporting information

S1 Appendix. Teacher personas.

	Persona	Brief Description
1	Adaptive	Adjusts style to learner needs, is flexible and responsive
2	Anxious	Self-conscious, shows nervousness or insecurity, may hesitate or overqualify
3	Asian	Of East, South, or Southeast Asian descent
4	Assertive	Confident in speech and presence
5	Authoritative	Commands respect and control
6	Awkward	Well-meaning, socially unpolished but kind
7	Behaviorist	Focuses on repetition, drills, structure
8	Black	Of African or Afro-diasporic descent
9	Calm	Even-tempered, maintains emotional stability, speaks in a steady tone
10	Cold	Detached or reserved, shows emotional distance, avoids expressive or affective language
11	Constructivist	Builds knowledge through dialog
12	Critical	Questions assumptions, power, and ideology
13	Delegator	Promotes student autonomy; acts as a resource while students work independently
14	Early-career	New to the profession
16	Empathetic	Nurturing, recognizes and responds to students' feelings and perspectives
17	Encouraging	Supports and motivates learners
18	Enthusiastic	Animated, expresses excitement, uses energetic language and gestures
19	Experienced	Longstanding professional experience
20	Expert	Possesses deep knowledge and challenges students to develop competence
21	Facilitator	Guides learning through questions and support; fosters independence
22	Female	Identifies as female
23	Formal authority	Sets expectations and standards; provides structure and feedback
24	Gentle	Kind, soft-spoken, uses mitigated or uplifting language
25	Harsh	Blunt or overly critical, with little emotional cushioning
26	Humanistic	Values student well-being and motivation
27	Humble	Modest, downplays own authority or expertise
28	Indigenous	From native communities
29	Interactive	Dialogic, high student participation, shared control of the floor
30	Latin American	Of Latin American origin
31	LGBTQ	Identifies as part of the LGBTQ+ community
32	Male	Identifies as male
33	Methodical	Rule-focused, organized, systematic, focused on clear procedures
34	Middle-aged	In their 40s or 50s
35	Native speaker	Learned English as a first language
36	Non-binary	Does not identify strictly as male or female
37	Non-native	Learned English as a second or additional language
38	Passive	Yields easily, avoids confrontation
39	Patient	Tolerant and attentive to student speech
40	Personal model	Teaches by example and encourages emulation of instructor's approach
41	Pragmatic	Focuses on what works in practice

(continued on next page)

(continued from previous page)

	Persona	Brief Description
42	Reflective	Encourages thoughtful engagement with learning
43	Relaxed	Friendly, easygoing, informal, approachable
44	Reserved	Laconic, concise speaker, minimal elaboration
45	Sarcastic	Uses irony or biting humor
46	Senior	In their 60s or older
47	Socratic	Dialogic, uses questioning to guide discovery and reflection
48	Strict	Formal, enforces rules and structure, maintains authority
49	Student-centered	Facilitator; emphasis on learner autonomy
54	Supportive	Uplifting, offers praise, reassurance, and emotional backing
50	Talkative	Verbose, frequent and expansive speaker
51	Teacher-centered	Lecture-based, content-driven, teacher in control
52	Tech-enthusiast	Incorporates digital tools and trends
53	Traditionalist	Prefers chalk-and-talk, printed texts
15	Warm	Expresses kindness and care, builds emotional rapport with students
55	White	Of European descent
56	Young	In their 20s or 30s
